# The Perils of Misspecified Priors and Optional Stopping in Multi-Armed Bandits

**DOI:** 10.3389/frai.2021.715690

**Published:** 2021-07-09

**Authors:** Markus Loecher

**Affiliations:** Berlin School of Economics and Law, Berlin, Germany

**Keywords:** multi-armed bandits, sequential testing, A/B testing, American options, optional stopping

## Abstract

The connection between optimal stopping times of American Options and multi-armed bandits is the subject of active research. This article investigates the effects of optional stopping in a particular class of multi-armed bandit experiments, which randomly allocates observations to arms proportional to the Bayesian posterior probability that each arm is optimal (*Thompson sampling*). The interplay between optional stopping and prior mismatch is examined. We propose a novel partitioning of regret into peri/post testing. We further show a strong dependence of the parameters of interest on the assumed prior probability density.

## 1 Introduction

Sequential testing procedures allow an experiment to stop early, once the available/streaming data collected is sufficient to make a conclusion. The benefits of reducing the duration of e.g. Web based A/B tests, clinical trials or the monitoring of adverse events are obviously very attractive in terms of saving costs and lives. Wald (Wald, 2004) proposed a sequential probability ratio test (SPRT) for continuous sequential analyses, where the observation ends if the likelihood ratio exceeds or falls below predetermined bounds. In order to reduce the dependence on the alternate hypothesis, Kulldorff et al. (Kulldorff et al., 2011) introduced the use of a maximized sequential probability ratio test (MaxSPRT), where the alternative hypothesis is composite rather than simple. A detailed comparison of (a modified) MaxSPRT and the O’Brien & Fleming test (O’Brien and Fleming, 1979) can be found in (Kharitonov et al., 2015). Note that all of these methods are typically limited to comparing two treatments at a time and deploy equal allocation, i.e. no attempt is made to maximize “rewards” during the testing phase. Multi-armed bandits (MABs) offer attractive solutions to both of these shortcomings.

One consequence of unplanned optional stopping rules for Null Hypothesis significance testing (NHST) are severely inflated type-I error rates. Bayesian hypothesis testing (BHT) is frequently hailed as a win-win alternative to traditional A/B testing. While there is a growing literature in favor of Bayesian stopping criteria based on the sequential collection of data (Rouder, 2014; Schönbrodt et al., 2017), other authors illustrate the dangers of optional stopping (Erica et al., 2014; Sanborn and Hills, 2014; de Heide and Grünwald, 2017; Hendriksen et al., 2018). This paper expands upon prior work (Loecher, 2017) and specifically focuses on the effects of optional stopping on the multi-armed bandit (MAB) procedure outlined by (Scott, 2010; Scott, 2012). In particular, we use simulations to demonstrate that the choice of the prior distribution has a significant impact on i) the power to detect differences between arms, ii) the average sample size as well as iii) the cumulative and terminal regret. In addition, we examine the precision of the final estimates and the dependence of the sample size on number of arms and effect sizes.

Our work touches upon previous research on American Options (Bank and Föllmer, 2003) that demonstrates a close connection between multi-armed bandits and optimal stopping times in terms of the Snell envelope of the given payoff process.

## 2 Multi-Armed Bandits

As compactly described in (Scott, 2015), the name multi-armed bandit (MAB) describes a hypothetical experiment where one faces k>1 slot machines (colloquially known as “one-armed bandits”) with potentially different expected payouts. In the typical setup there are k “arms,” a={1,…,k}, which of course are metaphors for simple Bernoulli experiments with success probability $p_{a}$. Arm a is associated with an unknown expected reward $\mu_{a}$ which for clarity we assume to be proportional to $p_{a}$. We further assume a Beta $\left(\alpha_{a},\beta_{a}\right)$ density as a prior distribution for $p_{a}$. The goal is two-fold: identify the arm with the greatest $p_{a}$ (or equivalently $\mu_{a}$ ) as soon as possible, and to accumulate the greatest total reward in doing so. This problem is similar to traditional sequential testing from the statistics literature (Wald and Wolfowitz, 1948; Jennison and Turnbull, 2000) but more complex due to the balancing of the so called explore/exploit dilemma: while one wants to find the arm with the highest reward, the total cost of the experiments needs to be minimized at the same time. The fundamental tension is between “exploiting” arms that have performed well in the past and “exploring” new or seemingly inferior arms in case their true performance is even better.

### 2.1 Randomized Probability Matching

Multiple algorithms have been proposed to optimize bandit problems: Upper Confidence Bound (UCB) methods (Lai and Robbins, 1985; Auer et al., 2002) for which strong theoretical guarantees on the regret can be proved, are very popular. The Bayes-optimal approach of Gittins (Gittins et al., 2011) directly maximizes expected cumulative payoffs with respect to a given prior distribution. Randomized probability matching (RPM), also known as Thompson Sampling (TS) (Thompson, 1933; Thompson, 1935), is a particularly appealing heuristic that plays each arm in proportion to its probability of being optimal. Recent results using Thompson sampling seem promising (Graepel et al., 2010; Granmo, 2010; Scott, 2010; May and Leslie, 2011; May et al., 2012), and the theoretical analysis is catching up (e.g. optimal regret guarantees for TS have been proven by (Agrawal and Goyal, 2012)).

In contrast to the fixed length, equal allocation of resources in NHST, RPM assigns the subsequent $n_{batch}$ samples in proportion to the ^1^posterior probability

$$\begin{array}{l} w_{a,t}\equiv P\left(\mu_{a}>\mu_{i\neq a}|Y_{a,t},N_{a,t}\right)=\\ {\int^{\text{​}}}_{0}^{1}\mathrm{Beta}\left(p_{a}|Y_{a,t}+\alpha_{a},N_{a,t}-Y_{a,t}+\beta_{a}\right)\left[\underset{j\neq a}{\prod }{\int^{\text{​}}}_{p_{j}}^{1}\mathrm{Beta}\left(p_{j}|Y_{j,t}+\alpha_{j},N_{j,t}-Y_{j,t}+\beta_{j}\right)dp_{j}\right]dp_{a} \end{array}$$

after each batch of recorded samples, where $Y_{a,t}$ and $N_{a,t}$ denote the cumulative number of successes and trials observed for arm *a* up to time *t* (which yield the observed sample proportions ${\hat{p}}_{at}=Y_{a,t}/N_{a,t}$). Thus, arm a∈{1,…,k} obtains $n_{a,t}=n_{batch}\cdot w_{a,t}$ samples on batch *t* respectively. This process is repeated until a set of stopping rules has been satisfied, the discussion of which we will defer to section 4. In this paper we do not allow for differences in prior parameters across arms, so from now on we assume $\alpha_{j}\equiv \alpha ,\beta_{j}\equiv \beta$.

### 2.2 Regret

It is worth noting that there are important differences between classical experiments and bandits w.r.t. the assumed optimality criteria. In many situations the “switching costs” between treatment arms with essentially equal rewards is small or even zero, which shifts all the costs to type-II errors. But even the concept of type II errors needs to be modified to properly reflect the loss function of a multi-armed bandit experiment:

1. Instead of “correctly rejecting a hypothesis” we care mainly about identifying the correct superior arm. For two arms, the concept of a *type S* and *type M* error (Gelman and Carlin, 2014) would be more appropriate.
2. For multiple (k>2) arms, there are k−1 different type-II errors.
3. The magnitude of the difference between the proclaimed and actual superior arm matters greatly.

A popular loss function which addresses all of the above concerns -at least for the testing phase-is regret, defined as the cumulative expected lost reward, relative to playing the optimal arm from the beginning of the experiment (Scott, 2010):

Let $a_{t}$ denote the arm of the bandit that was played at time *t* and $\mu_{a}$ denote the expected reward. Let $\mu^{\text{*}}=\max_{a}\left\{\mu_{a}\right\}$ be the expected reward under the truly optimal arm, and let $n_{a,t}$ denote the number of observations that were allocated to arm *a* at time *t*. Then the expected cumulative regret is

$$L_{T}^{c}=\overset{T}{\underset{t=1}{\sum }}L_{t}=\overset{T}{\underset{t=1}{\sum }}\underset{a}{\sum }n_{a,t}\left(\mu^{\text{*}}-\mu_{a}\right)$$

where we refer to the non cumulative $L_{t}$ as regret.

### 2.3 Choice of Priors

It is sometimes said that for MABs with Thompson sampling no tuning parameters have to be chosen at the onset of an experiment–unlike in classical testing where one has to fix the smallest difference to be detected at a certain confidence level in advance of the experiment.

We point out that the choice of the prior distribution for the binomial parameter plays a similar role to the parameter settings in NHST. For Gaussian priors (Honda and Takemura, 2014) showed that TS is vulnerable to prior misspecification, and (Bubeck and Liu, 2014) derive prior-free and prior-dependent regret bounds for more general cases.

In Sections 3, 4 we investigate the impact of a misspecified prior on the key metrics of a MAB experiment. Note that the consequences of a mismatch between the assumed and actual prior distribution have recently received a fair amount of attention in the literature (Rouder, 2014; de Heide and Grünwald, 2017; Hendriksen et al., 2018).

In the following simulation study (all simulations are run in R (R Core Team. R, 2016) using the bandit package (Lotze and Loecher, 2014)) we repeated each experiment 500 times with k=10 arms for a maximum of $N_{max}=1000$ periods. Each “batch” consisted of a total of $n_{batch}=k\times 50$ Bernoulli trials distributed across the *k* arms. We need to distinguish between two implementations of the prior distributions (each a beta distr. with parameters α,β):

1. Sampling the true values for the *k* binomial probabilities $p_{i}$. We analyze 2 “effect sizes”:
  a. “large”: $p_{i}$ concentrated in the range [0.05,0.14] (α=8.8,β=84).
  b. “small”: $p_{i}$ concentrated in the (4 times narrower) range [0.05,0.14] (α=61.5,β=941).
2. The assumed prior in the computation of the posterior. We analyze three types: i) a uniform (α=1,β=1), ii) a perfect match (assumed equal true prior), and iii) “strong disagreement” (a 3 times higher mean of the true prior than the assumed, equal variance).

Hence, we report on a total of 2×3 groups of experiments.

## 3 Infinite Experiments

In principle, bandits can run indefinitely, hence never forfeiting exploration, with the appealing consequence that the superior arms will eventually be correctly identified.

**FIGURE 1:**
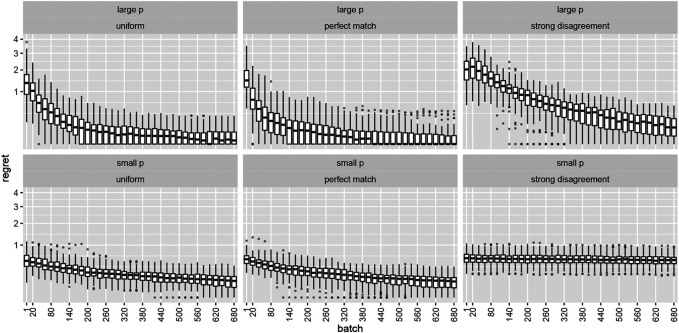
Boxplots depicting expected regret per time period $L_{t}$ for **(top row)** large and **(bottom row)** small effect sizes. The columns correspond to the three assumed priors outlined in the main text.

The top row of Figure 1 shows the temporal evolution of the distribution of the expected regret per time period $L_{t}$ for the case of relatively large effect sizes. With the exception of persistent outliers, the regret in the first two panels quickly decays to zero as sub-optimal arms are identified and decreasingly likely to receive further explorative samples. The rightmost panel illustrates the effects of a strong disagreement between the assumed and actual prior distribution; while the median regret also decreases, its interquartile range as well as the 95% percentile do not decrease. For the smaller effect size (bottom row), even the median regret does not seem to improve as time passes. Note the similarity of the results for the uniform and the perfectly matched prior which would support the perceived robustness of a non-informative prior. Maybe there is little to be gained (middle panels) but much to be lost (rightmost panels) if one tried to carefully calibrate the prior to the expected actual Bernoulli rates of the particular situation? We will address this question in the next section.

## 4 Stopped Experiments

In reality, infinitely long running experiments are rarely practical or even feasible. The purpose of experimentation is typically to identify superior treatments and eliminate inferior ones. We will discuss and implement two stopping rules both based on the posterior probability $w_{a,t}$ of arm *a* being optimal at time *t*. Of course, in practice, a maximum sample size is often enforced as a third termination criterion.

Rule I is to decide in favor of one arm as soon as its posterior probability crosses a threshold which we set to 0.95 and which is related to the power of the test, i.e. $w_{a,t}>0.95$. The second metric being monitored is the potential value remaining in the experiment, which is particularly useful when there are multiple arms. At any instance *t*, the arm with the maximum posterior probability, $a^{\text{*}}=argmax_{a}w_{a,t}$, is the most likely candidate to offer the highest success probability. The value remaining (VR) is the 95th percentile of the distribution of $\left(max\left({\hat{\mu }}_{a}\right)-{\hat{\mu }}_{a^{\text{*}}}\right)/{\hat{\mu }}_{a^{\text{*}}}$, where ${\hat{\mu }}_{a}$ are samples from the posteriors of arm(s) *a*. The VR attempts to estimate the amount of increased conversion rate^2^ one could get by choosing another arm, $a\neq a^{\text{*}}$. Note that the VR is a random variable with a distribution that depends on the observed data and is closely related to the value at risk metric ubiquitous in finance. Rule II (Scott, 2012) is to end the experiment when VR<0.01 ^3^. Rule III is to stop the experiments after $N_{max}=1000$ batches.

**FIGURE 2:**
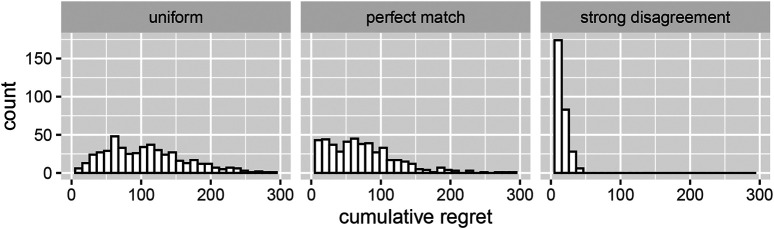
Cumulative expected regret under optional stopping for the large effect size only. The columns correspond to the three assumed priors outlined in Section 2.3 from the main text. The average cumulative regrets are 115,75,10, respectively.

For all three stopping rules, the arm with the largest posterior probability of being optimal is then chosen. Which metric should we use to evaluate the performance of this procedure? While it is tempting to simply take cumulative regret as a measure of loss as done in (Scott, 2010), this would ignore the quality of terminal decisions (It is easy to see that stopping early, i.e. reducing *T* would always decrease $L_{T}^{c}$!). In support of this claim, Figure 2 shows substantial “gains” for the case of the mismatched priors (cumulative regret is just 10). At the same time, we see that the average cumulative regret for the perfectly matched prior is substantially lower (75) than the one under the assumption of a uniform prior (115).

**FIGURE 3:**
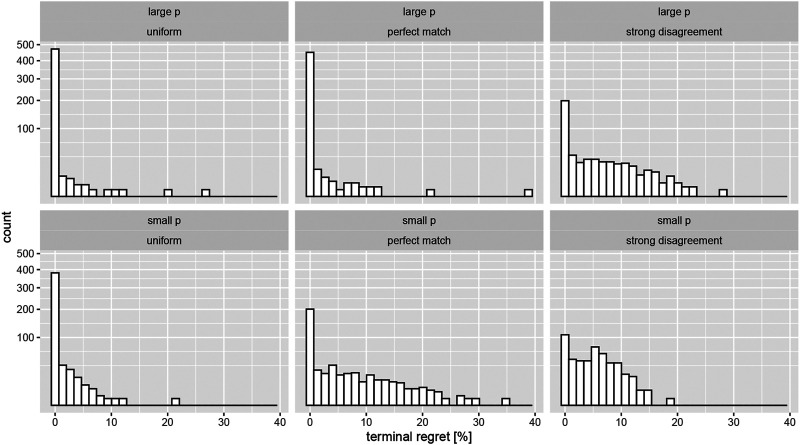
Terminal expected regret (measured in percent deviation from the true arm probability) under optional stopping for both effect sizes. The columns correspond to the three assumed priors outlined in the main text. Note the very large peaks at zero regret (sqrt scaling of the y axis).

Instead we adapt the train/test framework from machine learning to sequential testing and simultaneously monitor the expected regret after stopping the experiment. We define this terminal regret (TR) as the percent difference between the optimal arm and the one chosen by the bandit through a stopping rule: TR $=\left(\mu^{\text{*}}-\mu_{a^{\text{*}}}\right)/\mu^{\text{*}}$. We mention in passing its close similarity to the so-called simple regret defined in (Bubeck et al., 2011). The total cost of an experiment is a weighted sum of the cumulative and terminal regret, where the weights clearly depend on the ratio of added rewards during (peri) and after (post) testing. Figure 3 displays the distributions of the relative version of this metric which is most relevant for long running campaigns. As terminal regret is defined conditional upon having triggered the stopping rule, it is helpful to know the proportion of tests that did not terminate before the maximum test length, which is displayed in Table 1.

**TABLE 1:** Percent of tests which did not terminate before the maximum test length (=1000). Shown are results for the two effect sizes and the three priors.

	Uniform	Match	Disagreement
Large	25.4	16.9	0.0
Small	79.1	60.8	0.0

The numbers confirm the pattern gleaned from Figure 2; a strong mismatch of priors leads to overly aggressive test terminations, whereas the uniform prior results in a much more cautious decision making. We conclude this section with a possibly redundant message regarding the precision of the estimates of the superior arm.

**FIGURE 4:**
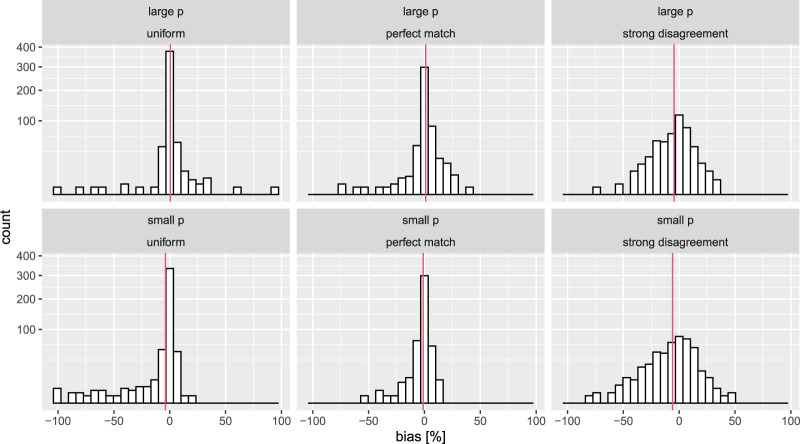
Variation of the bias in the estimate of the superior arm. The red vertical lines depict the empirical average of the bias.

Figure 4 shows the percent difference between the estimated arm probabilities at termination and the true one (for arm 10 which is always chosen to be the one with the largest reward). While there does not appear to be a noteworthy upward bias we again notice a somewhat strong dependence of the precision of the estimates and the assumed prior.

### 4.1 Calibration

While MABs make no guarantees on type-I/II errors, stopping rules I and II suggest precise probabilistic statements which a user might want to rely upon. We now show that the combination of optional stopping and mismatched priors leads to poor calibration of inferential expectations.

Table 2 displays the percent of correct early stopping decisions for each stopping rule, which should be close to 95% and seems to be correct for the case of agreeing priors (middle column). But the outcomes for the two other priors send a by now familiar message: either one faces overly aggressive test terminations (and hence inflated claims of posterior optimality probabilities) or the MAB is too conservative. The data in Table 3 complement the conditional nature of Table 2.

**TABLE 2:** Percent of correct early stopping decisions. Upper table shows results for rule I, lower table for rule II, as explained in the text. These fractions are conditional upon having triggered the stopping rule, the probabilities of which are shown in Table 3. For small effect sizes and a perfectly matching prior, a low value remaining is always declared before a significant posterior, which leads to the missing value in the 2nd row.

		Uniform	Match	Disagreement
Posterior >0.95	Large	100.0	95.0	48.7
	Small	80.0		21.3
Value <0.01	Large	98.4	96.4	49.2
	Small	94.1	96.2	27.2

**TABLE 3:** Percent of triggered early stopping decisions. Upper table shows results for rule I, lower table for rule II, as explained in the text. The column sums for corresponding rows in each table may add up to >100 as stopping rules are not mutually exclusive.

		Uniform	Match	Disagreement
Posterior >0.95	Large	5.0	16.1	55.0
	Small	1.0	0.0	67.6
Value <0.01	Large	74.6	83.1	100.0
	Small	21.0	39.2	100.0

### 4.2 Accuracy and ASN

All simulations up to this point have been “fully Bayesian” in the sense that the true *k* (fixed at k=10) arm probabilities were sampled from the corresponding prior distribution anew for each individual experiment. In this last section, we deviate from that strategy and instead consider equally spaced parameters in the interval [0.05−Δ;0.05] and vary k∈{2,5,10}, where the true effect size is then proportional to Δ. We further restrict ourselves to the uniform prior. Our goal is to find a dependence of accuracy and average sample number (ASN) on this effect size, which is somewhat more straightforward for this simpler experimental setup.

**FIGURE 5:**
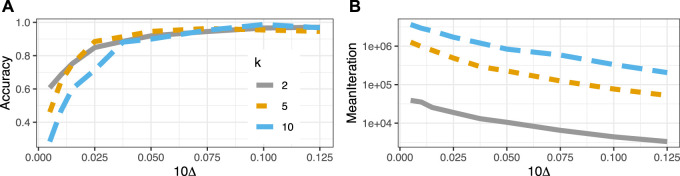
**(A)** Accuracy as a function of distance (Δ) to the reference arm 0.05. The true conversion probabilities for *k* arms are equally spaced in the interval [0.05−Δ;0.05]. **(B)** Average sample number (ASN) for multi armed bandits increases exponentially with shrinking distance (Δ) to the reference arm 0.05.

The left panel of Figure 5 shows the proportion of choosing the correct arm for a total of 2,5 and 10 arms respectively as a function of the distance Δ to the (optimal) reference arm $p_{0}=0.05$. For just two arms, the accuracy drops from the claimed 0.95 to 0.6 as Δ decreases from 0.01 to 0.0005 (Since the goal of experimentation is to find the optimal arm, error metrics from classification seem more suited than those from a traditional hypothesis testing framework; we therefore prefer accuracy over e.g. power). For multiple arms the loss of accuracy is much more dramatic and the onset earlier. The right panel of Figure 5 displays the average number of iterations/samples (ASN) the bandits took to make a decision. The logarithmic y-axis reveals an exponentially increasing effective sample size as the differences between the arms wane.

**FIGURE 6:**
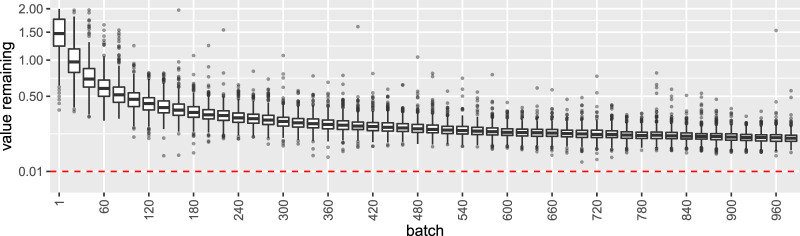
Distribution of the metric *value remaining* for the Null case of all k=10 arms being equal ($p_{i}=0.05$). With a uniform prior, not a single experiment ended earlier than the maximum number of iterations, i.e. the lower bound of 0.01 was never crossed!

We treat the special case of Δ=0 separately as a “Null simulation.” Surprisingly, with a uniform prior distribution, collecting $n_{batch}\times k\times N_{max}=50\times 10\times 1000$ samples does not generate sufficient evidence to declare a value remaining to be less than 1%, as Figure 6 shows.

## 5 Conclusion

Our contribution is to demonstrate a heightened sensitivity to prior assumptions in MABs when data-dependent stopping rules are used; a problem which has been known to be true in simple Bayesian inference for a few decades by now (Rosenbaum and Rubin, 1984). We have also shown that i) early high confidence decisions in MABs are much less reliable than claimed, and that ii) the prior parameters have a significant impact on most important metrics used to evaluate MABs. It seems that the burden of specifying tuning parameters has not vanished but is less directly obvious than in competing methods. Clearly, the sensitivity and expected sample size are determined by the beta prior parameters α,β. While the default uniform prior offers reasonable performance, it could be optimized by choosing more appropriate priors, at the risk of greatly worsening the outcome.

We further worry about the inability to easily set priors for the differences in Bernoulli probabilities rather than just the parameters themselves. The additional flexibility would allow to optimize for much more realistic experimental situations.

Of course, it is true that the cost of an experiment can be substantially reduced by deploying RPM based bandits instead of pre-committing to a fixed sample size. Allowing to rapidly detect large differences and not waste resources sticking to an unnecessarily rigid protocol is obviously a benefit of most sequential testing algorithms. One difference being that optimization attempted by Bayesian bandits is not concerned with a null hypothesis but with minimizing the posterior expected loss. The implicit assumption that Type-I errors incur no additional cost is likely to be wrong in realistic applications: it seems more reasonable that switching campaigns for no good reason should be avoided. These insights complement recent literature on frequentist-oriented bandit algorithms implementing optional stopping (Kharitonov et al., 2015; Johari et al., 2017; Aziz et al., 2018; Kaufmann et al., 2018).

## Data Availability

The original contributions presented in the study are included in the article, further inquiries can be directed to the corresponding author.

## Footnotes

1 which can easily be computed either by quadrature or simulation (see Figures 3, 4 in (Scott, 2010)). Note that alternatively (and computationally more efficient) we draw one random sample from each individual arm’s posterior and choose the one with the largest draw.

2 We use conversion rate synonymously to probability of success in a Bernoulli setting.

3 i.e. when there is a probability ≥0.95 that the value remaining is less than 1% of the current estimate of the highest conversion rate $p_{a^{\text{*}},t}$.
